# Syrian national growth references for children and adolescents aged 2–20 years

**DOI:** 10.1186/s12887-022-03331-0

**Published:** 2022-05-14

**Authors:** Ali Zamlout, Kamal Alwannous, Ali Kahila, Majd Yaseen, Raneem Albadish, Morhaf Aleid, Karina Hamzah, Mahmoud Monther, Oudai Akkari, Amah Hasan, Manal Hasan, Ammar Khallouf, Amjad Obied, Amna Schmidt, Sara Deeb, Orwa Deeb, Judie Jalal Eldin, Nour Ojaily, Mohammad Taifour, Qusai Ghanem, Younes Kabalan, Ali Alrstom, Marwan Alhalabi

**Affiliations:** 1 Department of Pediatric Surgery – Faculty of Medicine, Tishreen University, Latakia, Syria; 2 Department of Endocrinology and Metabolism, Al-Baath University, Homs, Syria; 3 Department of Dermatology and Venereology, Damascus University, 17th Nissan, Damascus, Syria; 4 Faculty of Medicine, Tishreen University, Latakia, Syria; 5 Faculty of Medicine, Damascus University, Damascus, Syria; 6 Faculty of Medicine, Al-Baath University, Homs, Syria; 7 Faculty of Medicine, Aleppo University, Aleppo, Syria; 8 Department of Endocrinology and Metabolism, Damascus University, Damascus, Syria; 9 Department of Infectious Diseases, Damascus University, Damascus, Syria; 10 Department of Reproductive Medicine, Embryology and Genetics, Damascus University, Damascus, Syria

**Keywords:** Growth references, Growth charts, Syria, Box-Cox T, Box-Cox Power Exponential, Box-Cox Cole and Green

## Abstract

**Background:**

During the past three decades, growth charts have become one of the principal tools for monitoring anthropometric development in individuals and populations as well. Growth references by the CDC and other countries have been widely used in our hospitals and healthcare units for clinical assessment of children’s development. The apparent overestimation and underestimation of many children's anthropometrics indicated the need to construct our own references. The objective of this study is to establish the national growth references for the Syrian population 2–20-year-old.

**Methods:**

A multicenter cross-sectional sample of 13,548 subjects, aged 2–20 years, were recruited from various kindergartens, schools, and universities across the Syrian Arab Republic between February and May-2019. Response variables (stature, weight, and BMI) were fitted against age using P-splines and three empirical distributions: Box-Cox T, Box-Cox Power Exponential, and Box-Cox Cole and Green. Residuals diagnostic Q-tests and worm plots were used to check the validity of fitted models.

**Results:**

Box-Cox T provided the best fit for stature-for-age, whereas Box-Cox Power Exponential provided the best fit for weight-for-age and BMI-for-age. Residuals diagnostics revealed adequate models fitting. BMI cutoffs revealed an increased prevalence of obesity (4.5% and 3.66%) and overweight (20.1% and 19.54%), for boys and girls respectively, in our population.

**Conclusions:**

Growth charts are available for use now in our hospitals and healthcare units. For 0–2-year-old children, we recommend using the World Health Organization’s standards.

**Supplementary Information:**

The online version contains supplementary material available at 10.1186/s12887-022-03331-0.

## Background

Somatic growth is the population-specific cumulative changes in height, weight, body mass index (BMI), and head circumference according to genetics, environmental, nutritional, and hormonal factors. It nearly follows a predictable phasic pattern, allowing healthcare providers to conduct practical assessments of child development and early detection of many growth disorders by using smoothed centiles [1, 2]. Since growth references are population-specific, several countries developed their charts and replicated them later to accommodate for the anthropometric changes in their populations over decades (secular changes or trends) [1, 3].

To date, growth references by the World Health Organization (WHO) have been used in our hospitals and healthcare units to track the growth of children under five years of age, while references by the Centers for Disease Control and Prevention (CDC) have been widely used in assessing older children. The apparent anthropometric misclassification resulting from the application of these references in the daily practice of pediatrics in our hospitals and outpatient clinics was a clear indication of the impracticality of relying on these references in assessing the growth of children in our population, and that it is important to develop our own national references for better growth assessments. Accordingly, the protocol for this research was developed by a group of professors and Ph.D. candidates at Damascus University, which in turn adopted the project and supervised the implementation of its steps in coordination with the Ministry of Health and the Ministry of Education in the Syrian Arab Republic. We herein present the growth reference charts for the Syrian population using the generalized additive models for location, scale, and shape (GAMLSS) [4].

## Methods

### Sample and procedure

The estimated Syrian population in 2020 is around 17.5 million, with a ratio of 100.22 males per 100 females, a life expectancy of 73.65 (years), a median age of 25.6 (years), an annual population growth of 2.49 (%), and a population density 95 (person/km2) [5]. We used a two-stage stratified sampling method to specify areas of which several kindergartens and schools would be randomly selected for sampling. In the first stage, the country was divided into five zones: Northern, Southern, Eastern, Western, and Central. Then a research team was assigned to each zone consisting of both graduate and senior medical candidates who were carefully trained in the steps of taking measurements and calibrating the instruments. In the second stage, each zone was divided into homogeneous sub-administrative areas (called strata). Schools/kindergartens in each stratum were selected by simple random sampling, and subsequently, all children in each selected school were measured – taking into account the size and socio-demographic characteristics of each stratum's population.

A multicenter cross-sectional sample of 13,572 subjects (49.5% males and 50.5% females), aged 2–20 years, were recruited from various kindergartens, schools, and universities across the Syrian Arab Republic between February and May-2019, under the supervision of Damascus university (ethical approval ID: 91–21-2–2019). The sampling framework was based on allocating the participants in uniformed sex-specific semi-annual groups between the ages of 2 and 20; each group consists of ≥ 160 participants, with oversampling in early childhood. The sample size and composition are consistent with Cole’s sampling guideline for constructing 0.4th-to-99.6th growth centiles [6].

Participants were asked to declare their approval to participate, medications, and any congenital or chronic disorders that may affect their growth; the kindergartens and school records for younger children were screened for that purpose. Participants were measured in the standing posture wearing minimal clothes and holding a deep breath, the head in the Frankfurt plane, the arms hanging loosely at their sides, the feet positioning slightly apart, and the back of the head, buttocks, and heels touching the vertical rod of the stadiometer (SECA – Germany). Two members checked the posture for each participant, and the third recorded the measurement to the last completed mm; also, two additional readings were recorded. Portable electronic weighing scales (SECA – Germany) were used for measuring weight to the nearest 100 g. All measurements were taken before 1 pm. Students who did not agree to participate were excluded from the measuring process.

### Data preparation

Age was converted to a fractional number of years between birthdate and date of measurement for each participant, without any grouping methods. The median of the three readings of stature was used in the analysis, and participants with measurements range > 1 cm were excluded from the study. The response variables for both sexes were plotted against age to visualize data distribution and any extreme values. Outliers that were apparently caused by data entry errors were returned to the audit team or excluded from the analysis if verification was not possible. A total of 13,548 participants were included in the statistical analysis. The BMI values were calculated using the relation (BMI = weight (kg)/stature2 (m2)).

### Statistical analysis

The LMS method was developed by Cole and Green [7, 8] to create centile curves for a response variable (e.g. stature) against a single explanatory variable (e.g. age), assuming that the response variable follows Box-Cox Cole and Green distribution, and a truncated standard normal distribution after Box-Cox power transformation. Rigby and Stasinopoulos generalized the LMS method to accommodate for kurtosis by introducing the Box-Cox Power Exponential [9] and Box-Cox T [10] distributions within the frame of GAMLSS.

Initial modeling for each response variable against age was applied using a combination of univariate penalized smoothing technique: P-spline [11], and three empirical distributions: Box-Cox T (BCT), Box-Cox Power Exponential (BCPE), and Box-Cox Cole and Green (BCCG). Models with the best fit were selected based on the Generalized Akaike’s Information Criterion (GAIC, with a penalty of # = 3) and Global Deviance scores [12]. Subsequently, an optimum power of transformation and degrees of freedom hyperparameters for the smoothers were obtained from the selected models, and adjusted for higher smoothness if needed. Hence, the growth curve at a time (t) can be summarized in up to four parameters: the approximate median (M), the approximate coefficient of variation (S), the skewness parameter (L), and the kurtosis parameter (T). To maximize the clinical utility of the charts, we used two-thirds of a standard deviation score as a channel width (the gap between two subsequent centiles) for selecting the set of centiles between 0.4 and 99.6 [13]. Finally, the models were interpolated to estimate the nine centiles (0.4th, 2.3th, 9th, 25th, 50th, 75th, 91st, 97.7th, and 99.6th) and LMS scores at each month between 2 and 20-year-old. The validity of the fitted models was checked using the residuals diagnostic Q-tests and worm plots for each one. The peak height velocity (PHV) was estimated by taking the first derivative of the median height curve for both sexes.

For BMI cutoffs, the equivalent z-scores to BMI values (30, 25, 18.5, 17, and 16 kg/m2) at age 18 were estimated and substituted into the centiles equation to select the corresponding centiles that pass through these values (relations are available in the original paper) [9]. Then, the centiles were plotted against the international cutoffs [14, 15] to visualize the difference between the curves.

## Results

**Fig. 1:**
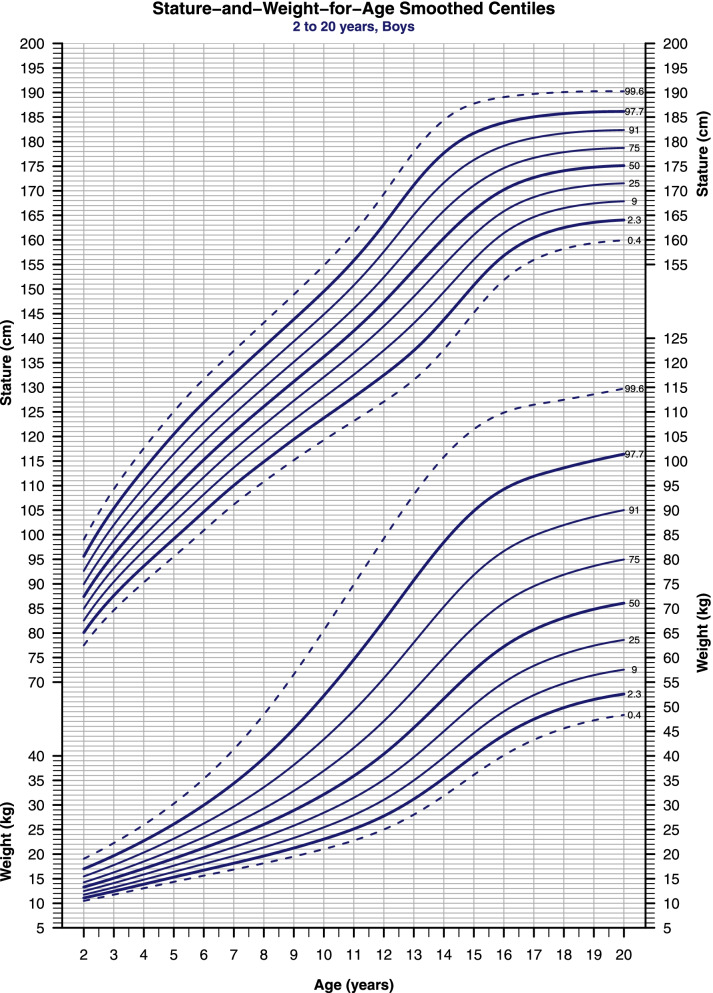
Stature-and-Weight-for-Age smoothed centiles for boys, 2–20-year-old. The extreme bold and dotted curves indicate the necessity for further investigations

**Fig. 2:**
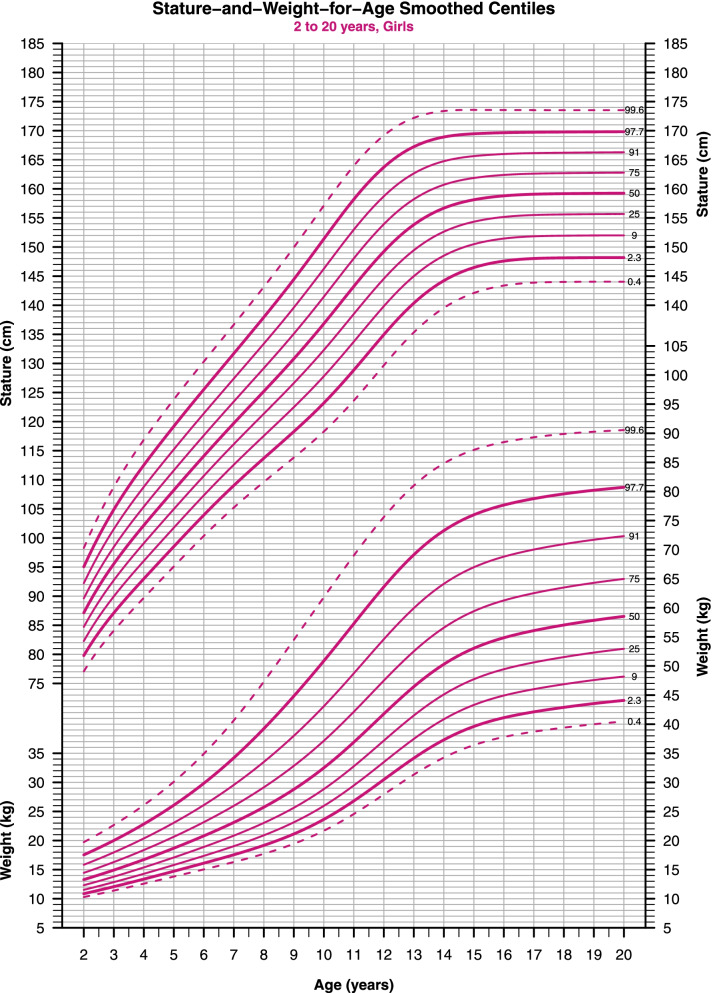
Stature-and-Weight-for-Age smoothed centiles for girls, 2–20-year-old. The extreme bold and dotted curves indicate the necessity for further investigations

**Fig. 3:**
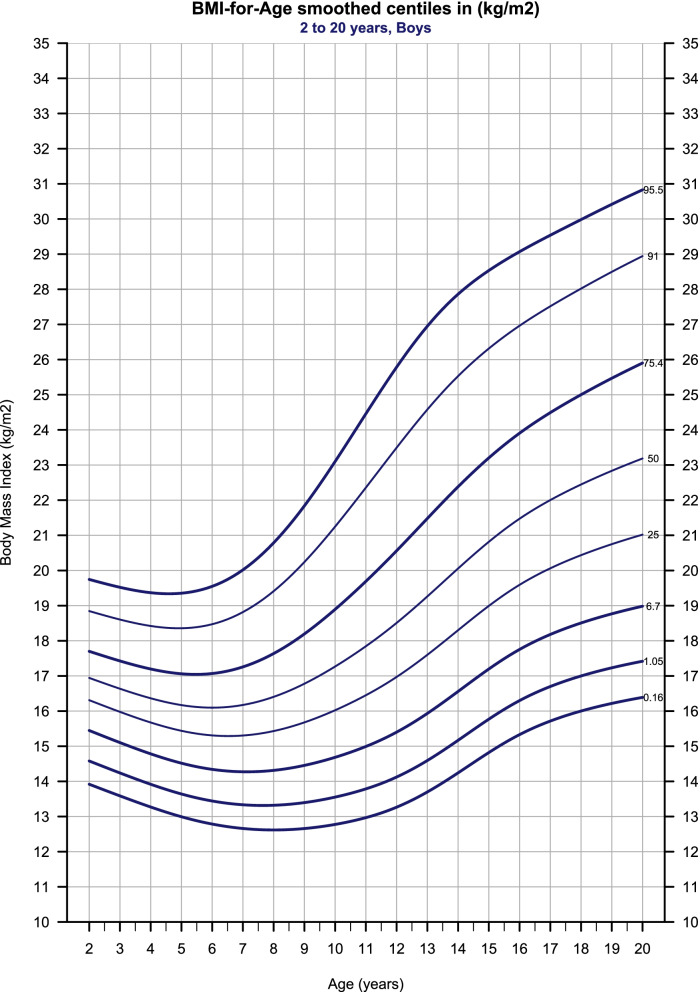
BMI-for-Age smoothed centiles for boys, 2–20-year-old. To ease the clinical utility of the chart, only the cutoffs centiles are presented – in addition to the 25^th^, 50^th^, and 91^st^ from the conventional centiles set. The bold curves represent the cutoffs for obesity, overweight, underweight grade -1, underweight grade -2, and underweight grade -3 respectively. The rest of the centiles are available in the full dataset (Supplementary Table 1)

**Fig. 4:**
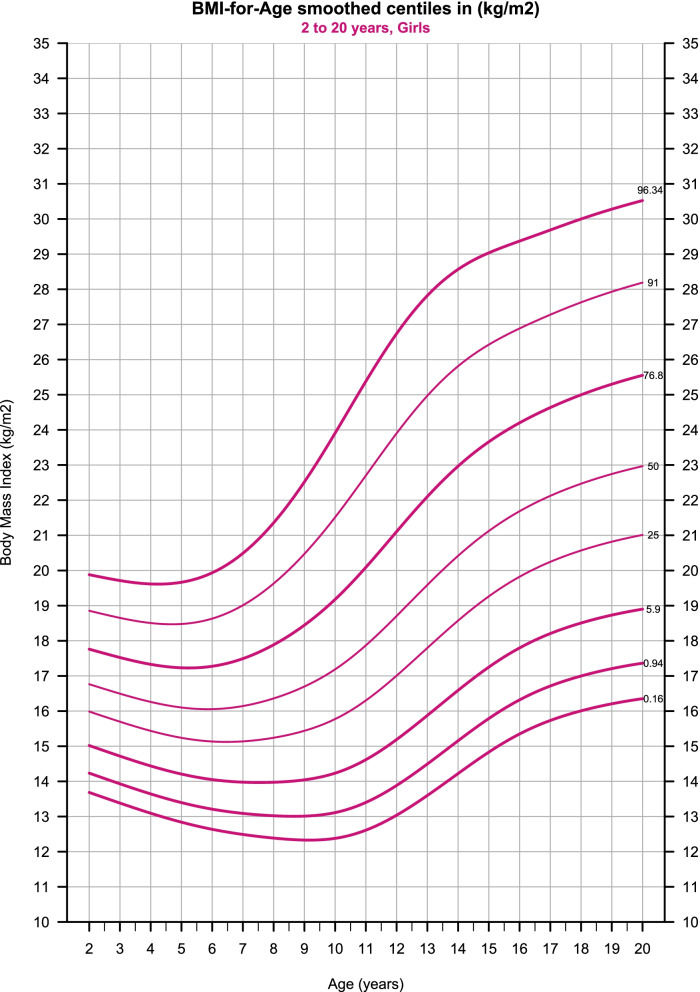
BMI-for-Age smoothed centiles for girls, 2–20-year-old. To ease the clinical utility of the chart, only the cutoffs centiles are presented – in addition to the 25^th^, 50^th^, and 91^st^ from the conventional centiles set. The bold curves represent the cutoffs for obesity, overweight, underweight grade -1, underweight grade -2, and underweight grade -3 respectively. The rest of the centiles are available in the full dataset (Supplementary Table 1)

A total of 13,548 (6,706 males and 6,842 females) students were measured and included in the statistical analysis, while 24 entries were excluded either due to a measurement range > 1 cm (n = 18) or outliers that we were unable to verify (*n* = 6). Among the three models, BCT provided the best fit for stature-for-age (Figs. 1, 2), whereas BCPE provided the best fit for weight-for-age and BMI-for-age (Figs. 1, 2, 3, 4). The residuals diagnostic worm plots and Q-statistics indicated adequate models fitting; the vast majority of points lay between the approximate 95% pointwise elliptical confidence bands, and p-values were greater than 0.05.

**Fig. 5:**
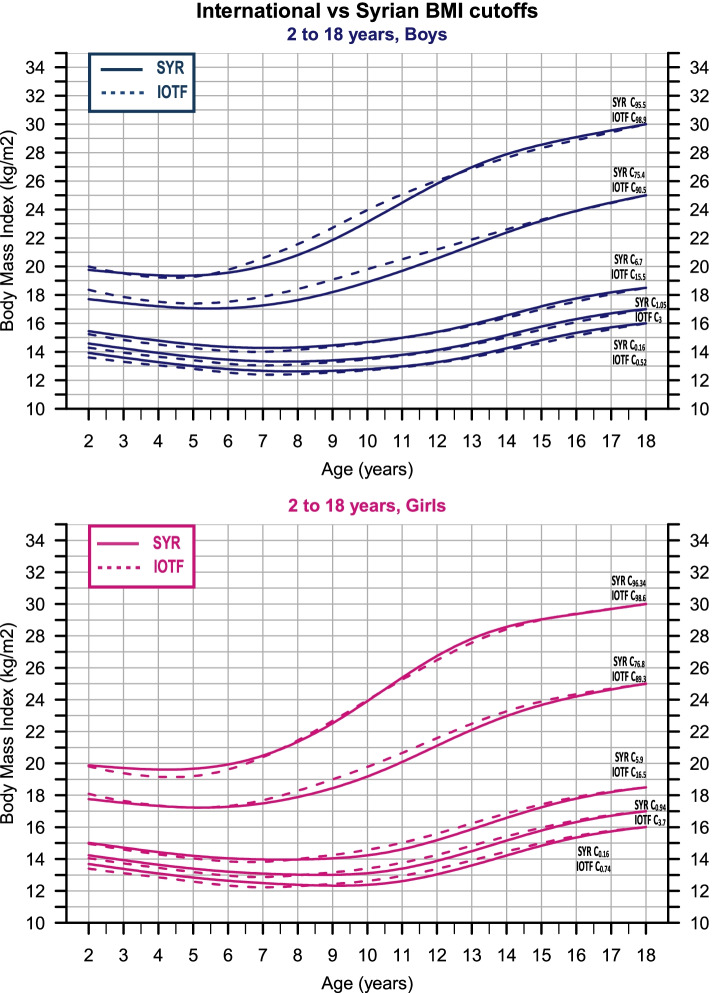
International (dotted) vs Syrian (solid) BMI cutoffs centiles, the discrepancy between the two sets is small

Centiles and LMS scores are presented in (Tables 2, 3, 4) against annual intervals of age; also, a full monthly set of the estimates is provided with this paper (Additional file 1). The age of maximum height increment was reached at age 13 (6.6 cm/year) for boys and 10.6 (6.48 cm/year) for girls. The prevalence of obesity estimated (4.5% and 3.66%), overweight (20.1% and 19.54%), underweight grade-1 (5.65% and 4.96%), underweight grade-2 (0.89% and 0.78%), and underweight grade-3 (0.16% and 0.16%) for boys and girls respectively. (Fig. 5 and Additional file 1) show the BMI centiles compared to the International cut-offs centiles.

## Discussion

Since its first appearance in the eighteenth century, the concept of studying human growth patterns has gained the attention of human biologists and public health experts [2]. This study provides the growth references for all children and adolescents in the Syrian population aging 2–20-year-old.

It is worth noting that, given the cross-sectional design of this study, centile crossing is not calibrated; thus, the resultant charts allows visualization of faster or slower growth velocities (a child’s growth curve that crosses the centiles up or down), but cannot quantify it [16].

### Stature for age

Parents tend to worry about short stature more than tallness; excessive growth in stature represents a rare complaint before puberty [17]. The selected set of centiles (0.4th-to-99.6th, spacing 0.67 SDS apart) provides more practical screening cutoffs than the conventional 3rd-to-97th centiles (Figs. 1, 2) [13, 18]. There is no consensus on specific thresholds to distinguish abnormal indices of stature. But with this centiles set, the 2.3th and 0.4th centiles (≈ -2 and -2.67 SD scores, respectively) provide a realistic decision region for an endocrinologist consultation and a better positive predictive value of screening tests for short stature [13]. The same goes for the 97.7th and 99.6th centiles (≈ + 2 and + 2.67 SD scores), but in clinical practice, children in this region are seldom diagnosed with growth disorders. See (Table 1) for further classification.

**Table 1:** Cutoffs and classifications for stature, weight, and BMI for age

Centile (SDS)	Stature	Weight	BMI
** > 99.6 (2.67)**	May be abnormal^b^	Overweight (Use BMI)^b^	Obesity^b^
** > 97.7 (2)**	May be abnormal^a^	May be abnormal (Use BMI)^a^	Obesity^b^
F > 96.34 (1.79) M > 95.5 (1.7)	Normal	Use BMI	Obesity^a^
** > 91 (1.33)**	Normal	Use BMI	Overweight^a^
F > 76.8 (0.73) M > 75.4 (0.69)	Normal	Use BMI	Overweight
** > 75 (0.67)**	Normal	Use BMI	Normal
**50 (0)**	Normal	Use BMI	Normal
** < 25 (-0.67)**	Normal	Use BMI	Normal
** < 9 (-1.33)**	Normal	Use BMI	Normal
M < 6.7 (-1.5) F < 5.9 (-1.56)	Normal	Use BMI	Thinness Grade-1
** < 2.3 (-2)**	Stunted^a^	Underweight^a^	Thinness Grade-1^a^
M < 1.05 (-2.31) F < 0.94 (-2.35)	Stunted^a^	Underweight^a^	Thinness Grade-2^a^
** < 0.4 (-2.67)**	Severely Stunted^b^	Severely Underweight^b^	Thinness Grade-2^b^
M < 0.16 (-2.95) F < 0.16 (- 2.95)	Severely Stunted^b^	Severely Underweight^b^	Thinness Grade-3^b^

*M* Males, *F* Females,

^a^Consider referring for further assessments

^b^Definitely should be referred to an endocrinologist. The table aims to ease the implementation of the charts in academic research and clinical practice by summarizing the guidelines presented in the paper

A simple way to predict a child’s stature in adulthood, with 95% confidence interval of about ± 9 cm, is to assume that the child’s centile will remain unchanged after the age of 2 years [1]. This prediction works better for children measured before puberty, and could be optimized by taking bone age into the account [1, 19]. Another approach is to adjust for parental stature, where the child’s target stature at age 20 is the mean of the biological parents’ stature (midparental stature) adjusted for sex (+ 7 cm for boys; and -7 cm for girls), with a range of 10 cm above and below this adjusted target stature [1]. The latter approach is useful to investigate relatively short children in tall families, where the target range represents the predicted child’s growth in family-conditioned centile terms, and deviating from this range indicates a growth disorder [1].

Secular stature trends are greater in childhood compared to adulthood because later generations are not only taller, but also more mature, than earlier generations of the same age [1]. Data from northern and southeastern Europe suggest an increment of stature up to 3 cm per decade [3, 20]. Unfortunately, estimating the secular trend of stature is not applicable in our case, as there are no previous studies on the Syrian population to compare with. (Table 2) summarizes the stature conditioned for age and sex in terms of centiles tabulated against annual intervals of age, see (Additional file 1) for monthly estimates.

**Table 2:** Stature-for-Age references for boys and girls

Age	Sex	L	M	S	C0.4	C2.3	C9	C25	C50	C75	C91	C97.7	C99.6
2	M	-0.3	87.4	0.042	77.5	80.1	82.5	85	87.4	89.9	92.6	95.6	99
3	M	-0.22	96	0.043	84.6	87.6	90.4	93.2	96	98.9	102	105.4	109.3
4	M	-0.14	102.9	0.045	90.3	93.6	96.7	99.8	102.9	106.2	109.6	113.3	117.6
5	M	-0.07	109.3	0.046	95.6	99.2	102.5	105.9	109.3	112.7	116.4	120.5	125.1
6	M	0	115.3	0.045	100.9	104.7	108.2	111.7	115.3	118.9	122.8	126.9	131.7
7	M	0.07	120.9	0.044	106.2	110	113.7	117.3	120.9	124.6	128.5	132.7	137.4
8	M	0.15	126.1	0.044	110.8	114.9	118.6	122.4	126.1	129.9	133.9	138.3	143.2
9	M	0.22	131.2	0.044	115.2	119.4	123.4	127.3	131.2	135.2	139.4	143.8	148.9
10	M	0.29	136.2	0.045	119.3	123.8	128	132.1	136.2	140.4	144.8	149.5	154.8
11	M	0.37	141.5	0.046	123.2	128	132.6	137	141.5	146	150.8	155.8	161.5
12	M	0.45	147.4	0.049	127.1	132.5	137.5	142.5	147.4	152.4	157.6	163.2	169.4
13	M	0.53	153.9	0.052	131.5	137.5	143	148.5	153.9	159.4	165.1	171.2	177.9
14	M	0.61	160.4	0.05	137.7	143.8	149.4	154.9	160.4	165.9	171.6	177.7	184.4
15	M	0.69	166	0.044	145.2	150.8	156	161	166	171.1	176.3	181.7	187.7
16	M	0.77	170.2	0.038	151.8	156.8	161.4	165.8	170.2	174.6	179.1	183.9	189.1
17	M	0.85	172.7	0.034	155.9	160.5	164.7	168.7	172.7	176.7	180.8	185	189.7
18	M	0.92	174.1	0.032	158.2	162.5	166.5	170.3	174.1	177.9	181.7	185.7	190.1
19	M	0.99	174.8	0.031	159.4	163.6	167.4	171.1	174.8	178.5	182.2	186	190.3
20	M	1.07	175.1	0.03	159.9	164.1	167.9	171.5	175.1	178.7	182.4	186.1	190.3
2	F	0.17	87.1	0.041	77.1	79.8	82.3	84.7	87.1	89.6	92.2	95	98.3
3	F	-0.1	95.6	0.043	84.1	87.1	90	92.7	95.6	98.5	101.5	104.9	108.8
4	F	-0.25	102.2	0.045	89.7	93	96.1	99.1	102.2	105.4	108.8	112.5	116.9
5	F	-0.31	108.2	0.045	95.1	98.5	101.8	105	108.2	111.6	115.2	119.2	123.8
6	F	-0.28	114.1	0.044	100.4	103.9	107.3	110.7	114.1	117.6	121.4	125.5	130.3
7	F	-0.19	119.7	0.045	105.2	109	112.6	116.1	119.7	123.4	127.4	131.7	136.7
8	F	-0.06	125.2	0.046	109.6	113.7	117.6	121.4	125.2	129.2	133.4	137.9	143.1
9	F	0.13	130.8	0.047	113.8	118.3	122.5	126.6	130.8	135.1	139.6	144.4	149.9
10	F	0.36	136.8	0.049	118.3	123.2	127.8	132.3	136.8	141.5	146.3	151.4	157.1
11	F	0.61	143.2	0.049	123.6	128.9	133.7	138.5	143.2	148	153	158.2	164
12	F	0.85	149.2	0.046	129.7	135	139.8	144.6	149.2	153.9	158.7	163.7	169.2
13	F	1.07	153.8	0.042	135.3	140.4	145	149.5	153.8	158.2	162.7	167.2	172.2
14	F	1.24	156.7	0.038	139.5	144.2	148.5	152.6	156.7	160.7	164.8	168.9	173.4
15	F	1.37	158.1	0.035	142.1	146.5	150.5	154.4	158.1	161.9	165.6	169.5	173.6
16	F	1.47	158.8	0.033	143.4	147.6	151.5	155.2	158.8	162.4	166	169.7	173.5
17	F	1.54	159.1	0.033	143.9	148	151.8	155.5	159.1	162.6	166.1	169.7	173.5
18	F	1.6	159.2	0.033	144	148.1	151.9	155.6	159.2	162.7	166.2	169.8	173.5
19	F	1.65	159.2	0.033	144	148.2	152	155.7	159.2	162.7	166.3	169.8	173.5
20	F	1.69	159.3	0.033	144	148.2	152	155.7	159.3	162.8	166.3	169.8	173.5

### Weight for age

The prevalence of obesity is increasing dramatically worldwide, leading to significant public health burdens and consequences [21]. On the other hand, underweight represents another problem in some countries [22]. Because of their public health importance, child adiposity should be routinely monitored in terms of weight and stature conditioned for age [21, 22]. We used the same set of centiles that were used in the stature-for-age charts; see (Figs. 1, 2).

There are no standard definitions of childhood obesity or underweight for use in weight-for-age charts [21], but similar cutoffs to those for the stature can be used for weight. The 2.3th and 97.7th centiles (≈ -2 and + 2 SDS) provide realistic cutoffs for further assessment of underweight and overweight respectively (see Table 1).

The efficiency of using weight charts independently of stature indices is limited, and weight should be adjusted for height to be evaluated properly [21, 22]. Although it is less sensitive than skinfold thickness [23], the BMI is a useful and widely used indicator of weight adjusted for height and age, and also provides the ability to standardize the cutoffs of overweight and thinness (discussed later) [21, 22].

Using a combination of the latter two approaches is a better practice in the clinical evaluation of child weight (For example, it may be possible to use weight-for-age charts to classify a child who weighs > 2(SD) above or below the corresponding population median as overweight or underweight, respectively. But it is difficult to classify children with weights < 2(SD) from the same median based on weight-for-age charts only, and the BMI-for-age charts in this case provide a better indicator of the adiposity as they take into account the child's height. Table 0.1 summarizes the guidelines for using both charts under different scenarios).

To simplify the implementation of this concept in daily clinical practice, TJ Cole [24] developed a “look-up” tool that can be added to the Stature-and-Weight-for-age charts (e.g., Figs. 1, 2) as a small graph, and it allows healthcare providers to predict the child's BMI centile without having to use the BMI-for-age chart. Thus, it is possible to assess the three anthropometric measurements together using only one sheet of paper.

(Table 3) summarizes the weight references conditioned for age and sex in terms of centiles tabulated against annual intervals of ages, the monthly estimates are available within the (Additional file 1).

**Table 3:** Weight-for-Age references for boys and girls

Age	Sex	L	M	S	C0.4	C2.3	C9	C25	C50	C75	C91	C97.7	C99.6
2	M	-1.41	13.3	0.103	10.5	11.1	11.7	12.5	13.3	14.3	15.5	17	19.1
3	M	-1.38	15.1	0.11	11.7	12.4	13.2	14.1	15.1	16.3	17.8	19.7	22.3
4	M	-1.34	17.1	0.118	13.1	13.9	14.8	15.9	17.1	18.5	20.3	22.7	26
5	M	-1.3	19.1	0.127	14.4	15.3	16.4	17.7	19.1	20.9	23.1	26.1	30.3
6	M	-1.25	21.3	0.139	15.6	16.7	18	19.5	21.3	23.4	26.2	30	35.3
7	M	-1.19	23.5	0.152	16.9	18.1	19.6	21.4	23.5	26.2	29.7	34.4	41.3
8	M	-1.12	26	0.166	18.1	19.6	21.4	23.5	26	29.3	33.6	39.5	48.4
9	M	-1.03	28.9	0.181	19.5	21.2	23.3	25.8	28.9	32.9	38.2	45.5	56.6
10	M	-0.93	32.2	0.195	21	23	25.5	28.4	32.2	37	43.4	52.2	65.6
11	M	-0.82	35.9	0.207	22.8	25.1	28	31.5	35.9	41.6	49.2	59.6	74.9
12	M	-0.72	40.4	0.215	25	27.8	31.1	35.2	40.4	47.1	55.8	67.5	84.2
13	M	-0.63	45.7	0.217	28	31.2	35	39.7	45.7	53.3	63.1	75.7	93.2
14	M	-0.57	51.6	0.211	31.9	35.4	39.7	45	51.6	60	70.4	83.5	100.8
15	M	-0.53	57.4	0.2	36.1	40	44.7	50.3	57.4	66.2	76.8	89.8	106.5
16	M	-0.51	62.2	0.189	40.1	44.2	49	54.9	62.2	71.1	81.7	94.3	109.9
17	M	-0.5	65.7	0.178	43.3	47.5	52.4	58.3	65.7	74.5	84.8	96.9	111.5
18	M	-0.5	68.1	0.171	45.6	49.8	54.7	60.7	68.1	76.9	87	98.6	112.5
19	M	-0.5	69.8	0.167	47.2	51.5	56.4	62.4	69.8	78.6	88.7	100.1	113.6
20	M	-0.5	71.1	0.165	48.3	52.6	57.5	63.6	71.1	80	90	101.4	114.7
2	F	-1.36	13.3	0.116	10.3	10.9	11.5	12.3	13.3	14.4	15.8	17.5	19.7
3	F	-1.3	14.9	0.123	11.4	12.1	12.9	13.8	14.9	16.3	18	20	22.7
4	F	-1.24	16.7	0.13	12.6	13.4	14.3	15.4	16.7	18.4	20.4	22.8	26.1
5	F	-1.19	18.7	0.138	13.8	14.7	15.8	17.1	18.7	20.6	23.1	26.1	30.1
6	F	-1.13	20.8	0.149	15	16.1	17.4	18.9	20.8	23.2	26.1	29.8	34.9
7	F	-1.08	23.1	0.161	16.3	17.6	19	20.8	23.1	26	29.6	34.2	40.7
8	F	-1.02	25.7	0.172	17.7	19.2	20.9	23	25.7	29.2	33.5	39.2	47.3
9	F	-0.96	28.8	0.181	19.4	21.1	23.1	25.6	28.8	32.8	38	44.8	54.5
10	F	-0.9	32.5	0.185	21.7	23.6	26	28.8	32.5	37.1	43.1	50.9	61.8
11	F	-0.84	36.9	0.185	24.6	26.8	29.5	32.8	36.9	42.1	48.7	57.3	69
12	F	-0.79	41.8	0.18	27.9	30.5	33.5	37.2	41.8	47.5	54.6	63.6	75.6
13	F	-0.74	46.4	0.173	31.3	34.2	37.5	41.5	46.4	52.5	59.9	69.1	81
14	F	-0.69	50.3	0.167	34.3	37.3	40.8	45.1	50.3	56.6	64.1	73.3	84.8
15	F	-0.64	53	0.161	36.4	39.6	43.3	47.7	53	59.4	67	76	87.1
16	F	-0.59	54.9	0.158	37.8	41.1	44.9	49.4	54.9	61.3	68.8	77.7	88.5
17	F	-0.55	56.1	0.156	38.7	42.1	46	50.6	56.1	62.5	70	78.8	89.3
18	F	-0.5	57	0.154	39.4	42.9	46.9	51.5	57	63.5	70.9	79.6	89.9
19	F	-0.46	57.8	0.152	40	43.5	47.6	52.3	57.8	64.3	71.7	80.2	90.3
20	F	-0.42	58.5	0.151	40.5	44.1	48.2	53	58.5	65	72.3	80.7	90.5

### Body-mass-index for age

The Body Mass Index (weight/height2) represents a special form of the weight/height(p) index, where p is fixed at 2 instead of varying with age [25]; thus, it became a widely used indicator throughout infancy, childhood, adolescence, and adulthood [22]. Clinically, the BMI charts are used in the same way as stature and weight ones, where single measurements are plotted on the chart, and extreme estimates or marked centile crossing indicate the need for further assessments [1].

The dramatic secular trend of increasing body fatness in recent decades led to global concerns about childhood obesity and its consequences in adulthood [26, 27], with several incompatible definitions for overweight and obesity [15]. In 2000, the International Obesity Task Force (IOTF) used BMI data from six countries to standardize the definition for child overweight and obesity, defining a BMI of 25 and 30 (kg/m2) at age 18 as cutoffs for overweight and obesity respectively [21]. TJ Cole et al. [22] extended these international cutoffs to include thinness, defining a BMI of 18.5, 17, and 16 (kg/m2) at age 18 as cutoffs for thinness grade 1, 2, and 3, respectively. Both approaches used the LMS method to establish country-specific centiles passing through the mentioned values, and subsequently averaged the centiles to estimate the cutoffs [21, 22]. Recently, Cole et al. updated this methodology by averaging the LMS curves instead of the centiles, which allowed for expressing the cutoffs in centile terms [15].

A noteworthy limitation of these cutoffs is that they did not take into account data from low-income countries or countries in Africa and the Middle East; the authors assumed that the cutoffs are valid to use worldwide though, and emphasized the importance of testing these cutoffs against new data [22]. Since the case of Syria fulfills both conditions, it is an appropriate moment to test this assumption against our data. We used the same approach as IOTF to select the centiles passing through the aforementioned BMI values at age 18. The difference between our centiles and the international ones, compared to the centiles used to establish the latter, is small (Fig. 5). Our results support the assumption that these cutoffs are suitable for use internationally and encourage other countries to use them.

Another advantage of the international BMI definitions is the ability to estimate the prevalence of obesity, overweight, and thinness in the population of interest. It is a little bit surprising to observe such a high prevalence of overweightness (20.1% and 19.54%) in our children after eight years of war and food shortages, compared to Middle Eastern countries such as the UAE (15.3% and 16.1%) or the recent pooled estimates by the IOTF (8.4% and 9.3%) for boys and girls, respectively [15, 28]. However, this increase can be attributed to a combination of factors: (A) The global pandemic of obesity, recent studies indicate a rapid expansion in obesity and overweight categories [29, 30]; (B) The rapid deterioration of socio-economic status and its association with increased prevalence of overweight in developing countries [31, 32]; (C) Switching to high-carbohydrate diets in light of food shortages and declining household financial income. There are no clear boundaries between these factors, but the overall effect is an increase in the prevalence of overweightness, which calls for effective intervention by the government to study this problem and deal with it.

(Table 4) summarizes the BMI references conditioned for age and sex in terms of centiles tabulated against annual intervals of ages. To simplify the use of the BMI charts in clinical practice (Table 1), we used the cutoff centiles in addition to the 25th, 50th, and 91st centiles (Figs. 3, 4). The rest of the centiles were provided within the full monthly dataset (Additional file 1) and can be plotted with any statistical software.

**Table 4:** BMI-for-Age references for boys and girls

**Age**	**Sex**	**L**	**M**	**S**	**C0.16**	**C1.05**	**C6.7**	**C25**	**C50**	**C75.4**	**C91**	**C95.5**
2	M	-2.86	16.9	0.072	13.9	14.6	15.4	16.3	16.9	17.7	18.8	19.7
3	M	-2.75	16.6	0.076	13.6	14.2	15.1	16	16.6	17.4	18.6	19.5
4	M	-2.65	16.4	0.079	13.3	13.9	14.8	15.7	16.4	17.2	18.4	19.4
5	M	-2.55	16.2	0.084	13	13.6	14.5	15.4	16.2	17.1	18.4	19.4
6	M	-2.44	16.1	0.09	12.8	13.4	14.3	15.3	16.1	17.1	18.5	19.5
7	M	-2.34	16.2	0.098	12.7	13.3	14.3	15.3	16.2	17.3	18.8	20
8	M	-2.23	16.4	0.108	12.6	13.3	14.3	15.4	16.4	17.6	19.4	20.8
9	M	-2.13	16.8	0.118	12.7	13.4	14.5	15.7	16.8	18.2	20.2	21.8
10	M	-2.03	17.3	0.129	12.8	13.6	14.7	16	17.3	18.9	21.2	23.1
11	M	-1.92	17.8	0.139	13	13.8	15	16.5	17.8	19.7	22.4	24.5
12	M	-1.82	18.5	0.146	13.3	14.1	15.4	17	18.5	20.6	23.5	25.8
13	M	-1.71	19.3	0.15	13.7	14.6	15.9	17.6	19.3	21.5	24.6	26.9
14	M	-1.61	20.1	0.15	14.2	15.2	16.6	18.3	20.1	22.4	25.5	27.9
15	M	-1.51	20.8	0.148	14.8	15.8	17.2	19	20.8	23.2	26.3	28.5
16	M	-1.4	21.5	0.145	15.3	16.3	17.8	19.6	21.5	23.9	27	29.1
17	M	-1.3	22	0.144	15.7	16.7	18.2	20.1	22	24.5	27.5	29.5
18	M	-1.2	22.4	0.145	16	17	18.5	20.4	22.4	25	28	30
19	M	-1.09	22.8	0.146	16.2	17.2	18.8	20.7	22.8	25.5	28.5	30.4
20	M	-0.99	23.2	0.147	16.4	17.4	19	21	23.2	25.9	28.9	30.8
**Age**	**Sex**	**L**	**M**	**S**	**C0.16**	**C0.94**	**C5.9**	**C25**	**C50**	**C76.8**	**C91**	**C96.34**
2	F	-2.07	16.8	0.079	13.7	14.2	15	16	16.8	17.8	18.9	19.9
3	F	-2.05	16.5	0.082	13.4	13.9	14.7	15.7	16.5	17.5	18.7	19.7
4	F	-2.03	16.3	0.086	13.1	13.6	14.4	15.4	16.3	17.3	18.5	19.6
5	F	-2	16.1	0.091	12.8	13.4	14.2	15.2	16.1	17.2	18.5	19.7
6	F	-1.95	16.1	0.097	12.6	13.2	14.1	15.1	16.1	17.3	18.6	19.9
7	F	-1.89	16.1	0.106	12.5	13.1	14	15.1	16.1	17.5	19	20.5
8	F	-1.82	16.4	0.117	12.4	13	14	15.2	16.4	17.9	19.6	21.4
9	F	-1.73	16.7	0.129	12.3	13	14	15.4	16.7	18.4	20.5	22.5
10	F	-1.64	17.2	0.141	12.4	13.1	14.2	15.8	17.2	19.2	21.5	23.9
11	F	-1.53	17.9	0.151	12.6	13.4	14.6	16.3	17.9	20.1	22.7	25.4
12	F	-1.43	18.7	0.155	13	13.9	15.2	17	18.7	21.1	23.9	26.7
13	F	-1.34	19.6	0.155	13.6	14.5	15.9	17.8	19.6	22.1	25	27.8
14	F	-1.27	20.4	0.152	14.2	15.2	16.6	18.6	20.4	23	25.8	28.6
15	F	-1.21	21.1	0.147	14.8	15.8	17.3	19.3	21.1	23.7	26.4	29
16	F	-1.17	21.7	0.142	15.3	16.3	17.8	19.8	21.7	24.2	26.9	29.4
17	F	-1.13	22.1	0.139	15.7	16.7	18.2	20.2	22.1	24.6	27.3	29.7
18	F	-1.09	22.5	0.138	16	17	18.5	20.6	22.5	25	27.6	30
19	F	-1.05	22.7	0.138	16.2	17.2	18.7	20.8	22.7	25.3	27.9	30.3
20	F	-1.02	23	0.138	16.4	17.4	18.9	21	23	25.6	28.2	30.5

### Puberty and Peak Height Velocity (PHV)

Puberty is a series of complex events in the primary and secondary sexual characteristics following the maturation of the hypothalamic-pituitary–gonadal axis, with a wide variation between individuals in timing and tempo [33]. Recent studies indicate a pubertal onset between the ages of 9 and 14 years in boys and 8 and 13 years in girls [34]. The pubertal growth spurt, where growth velocity raises to a peak (PHV) and then tail-off in adulthood, represents a key-feature within the process of puberty. We estimated the age at PHV in both sexes by taking the first derivative of the median height curve; PHV was reached at the age of 13 (6.6 cm/year) in boys and 10.6 (6.48 cm/year) in girls. In comparison with estimates from Turkey (13.7 and 11.3 years) [35, 36], the Syrian population seem to be relatively more advanced in pubertal timing, and much closer to the Saudi (13.5 and 10.5 years)[37] and Emirati (13 and 11 years)[28] populations for boys and girls, respectively.

It is worth noting that deriving the age at PHV from cross-sectional data is unbiased but may result in lower increment values compared to longitudinal data, as a recent paper showed [38].

### Limitations and strengths

This study was conducted after eight years of a war that comprised potential socioeconomic and nutritional constraints; the lack of self-motivation, insufficient time to exercise, and switching toward high-carbohydrates diets could be implicated in the increased prevalence of overweight. Skinfold thickness and waist or mid-arm circumference would have provided a better insight into that problem as they increase the sensitivity of BMI in evaluating obesity, but unfortunately, they were not included in our protocol from the beginning. Another limitation regarding the war is that some northeastern regions of the country were not accessible during the measurement phase of this study, and we have no data from them.

For a long time, the lack of national growth references has been an obstacle to numerous studies on growth, malnutrition, and obesity in Syria. It is now possible for healthcare providers to evaluate children's development and make objective clinical decisions more accurately. The new set of centiles used in this study (0.4th-to-99.6th, spacing 0.67 SDS apart) is compatible for use with Cole's tool [24], more accurate to implement in clinical practice and screening tests [13, 18], and easier to build upon [24]. There is a trend to unify the use of this set in the next generations of growth charts [13]. Although the paper was published after the measurement phase of our study ended, but the sample size and composition are consistent with Cole’s guideline for constructing growth references, given the selected set of centiles [6]. We emphasize the importance of using this guideline in future studies as it provides a genuine basis for sampling frameworks.

Syria's location in the middle of three continents (Europe, Asia, and Africa), in addition to its classification as a low-income country, formed distinct conditions to investigate the validity of using the international BMI cutoffs regardless of race or origin; our results support this assumption.

Although the war and its nutritional and socioeconomic impacts, the findings reveal that Syria is not isolated from the global obesity pandemic, which calls for efficient governmental intervention to reduce the problem and opens the door to many questions related to nutrition and public health interventions during humanitarian crises. Finally, neighboring countries with similar environmental and socioeconomic conditions may be able to use our charts until they develop their own references.

## Conclusion

Growth reference charts for the Syrian population 2–20-year-old are now available for use in our hospitals and healthcare units. Doctors should understand the statistical background of these charts, their limitations and strengths, and how to use them properly in the light of global and local secular changes. The IOTF project should have international support as it provides a very reasonable solution for countries that have not established their growth references. Growth at 0–2 years of age is much more complex, and unlike older ages, centile crossing is a natural finding [1]; we recommend using the WHO’s growth standards for this category [39]. We also recommend the use of Cole’s guideline for constructing future growth reference centiles [6].

## Supplementary Information

**Additional file 1.** Stature, Weight, and BMI for Age centiles.

## Data Availability

According to the protocol signed between the authors and Damascus University, the data is legally owned by the latter and is available upon reasonable request by contacting the Dean’s office of the Faculty of Human Medicine: info@damascusuniversity.edu.sy.

## Abbreviations

LMS: Lambda-Mu-Sigma method
BMI: Body Mass Index
PHV: Peak Height Velocity
BCT: Box-Cox T
BCPE: Box-Cox Power Exponential
BCCG: Box-Cox Cole and Green
IOTF: International Obesity Task Force
GAMLSS: Generalized Additive Models for Location, Scale, and Shape
